# Prophylactic Effects of Combined Bosentan and Nintedanib on Early Post-Traumatic Joint Contracture Formation in a Rat Model

**DOI:** 10.3390/jcm15114116

**Published:** 2026-05-26

**Authors:** Erik Wegner, Dennis Warnke, Victoria Buschmann, Alexander Pirkl, Martin Betz, Ulrike Ritz, Tara S. Mühlschlegel, Sven-Oliver Dietz, Erol Gercek, Andreas Baranowski

**Affiliations:** 1 Biomatics Group, Department of Orthopaedics and Traumatology, University Medical Center of the Johannes Gutenberg University, 55131 Mainz, Germany; 2 Wharton School, University of Pennsylvania, Philadelphia, PA 19104, USA; 3 Department of Trauma Surgery, Orthopaedics and Reconstructive Surgery, ANregiomed Hospital, 91522 Ansbach, Germany

**Keywords:** bosentan, nintedanib, arthrofibrosis, joint contracture, translation, myofibroblast, PTJC

## Abstract

**Background/Objectives**: Post-traumatic joint contracture (PTJC) is a common complication in orthopedic surgery characterized by fibrosis and restricted joint mobility. Previous work by our multi-arm study demonstrated that nintedanib monotherapy could effectively mitigate PTJC by targeting fibroblast activation pathways. Since PTJC involves multiple simultaneously activated signaling pathways, we hypothesized that combination therapy targeting both inflammation and fibrosis might be advantageous for improving outcomes. As a candidate for combination treatment with nintedanib, bosentan, an endothelin receptor antagonist that modulates inflammatory responses, was chosen to assess whether this approach could improve the prevention of PTJC. **Methods**: Thirty-nine Sprague Dawley rats were randomized evenly into three groups after undergoing a standardized hyperextension trauma of the knee and K-wire arthrodesis over a period of two weeks. The experimental groups (*n* = 13 each) received a combination (COMB) of nintedanib (5 mg/kg/d, twice daily) and bosentan (50 mg/kg/d, twice daily), or nintedanib (5 mg/kg/d, twice daily) alone throughout the immobilization period. The control group (*n* = 13), meanwhile, was given a placebo. Joint mobility was evaluated quantitatively by measuring the contracture angle (CA) and resistance to extension. Additionally, tissue from the posterior joint capsule was collected for histological analysis. Quantitative PCR was used to analyze tissue and assess the expression levels of genes involved in the pro-fibrotic process, including, *Il-6*, *Tgf-β*, *Nf-κb*, *Ctgf*, and *α-Sma*. Statistical analysis involved Kruskal–Wallis and ANOVA tests with post hoc methods, with significance defined at *p* < 0.05. **Results**: Both combination therapy and nintedanib monotherapy significantly reduced the contracture angle compared to placebo (*p* ≤ 0.05 and *p* ≤ 0.01, respectively), with no significant difference between treatments. Histological analysis showed significantly fewer myofibroblasts in the COMB and nintedanib groups versus placebo (*p* < 0.05). α-SMA expression was significantly decreased by 8-fold (COMB) and 11-fold (nintedanib) compared to placebo (*p* < 0.05), with no differences between treatments. No significant differences were detected in upstream pro-fibrotic gene expression among groups. **Conclusions:** Based on results comparable to those achieved with nintedanib monotherapy, the additional administration of bosentan does not appear to offer any further benefit at the given experimental setup.

## 1. Introduction

Wound healing and fibrosis represent closely related biological processes. These two processes, initiated by injury, are orchestrated by the same cellular types and share many mechanisms that are fundamentally similar in all tissues. Fibrosis is characterized by a disruption in the otherwise precise and self-limiting interplay of these mechanisms. This results in an imbalance between the formation and degradation of connective tissue, leading to its accumulation with subsequent organ failure [1,2,3]. Across all tissues, the course of fibrosis is organized into temporally overlapping phases [2,4]. Injury causes oxidative stress and an excessive inflammatory response, which includes the release of an array of proinflammatory cytokines and pro-fibrogenic TGF-β. In association with cells of the innate and adaptive immune systems, fibroblasts and macrophages are activated and enabled to produce excessive amounts of connective tissue components [1,5].

Post-traumatic joint contracture (PTJC), one of the most common complications following orthopedic joint surgery and trauma, is considered a fibrotic condition [5,6]. Intra-articular connective tissue proliferation and peri-articular adhesions result in restricted and painful mobility of the joint. The impact of PTJC on the large joints can profoundly lower the quality of life and autonomy of those affected [7,8]. Conventional therapy methods for PTJC have historically centered on rehabilitation and surgery to address existing restrictions. However, these methods are rarely crowned with success and entail the risk of re-traumatization, which can lead to a significant exacerbation of the PTJC [5,8]. Pharmacological correction of aberrant, pro-inflammatory, or pro-fibrotic signaling cascades in the early stages of PTJC could be an elegant alternative to conventional therapy [5]. In the absence of formally recognized agents for this condition, the role of preventative pharmacotherapy remains marginal [5,8,9].

Substantial progress in understanding the underlying pathomechanisms of other fibrotic conditions has led to the development and approval of nintedanib, a tyrosine kinase inhibitor that primarily interferes with fibroblast activation pathways by inhibiting platelet-derived growth factor (PDGF), fibroblast growth factor (FGF), and vascular endothelial growth factor (VEGF) receptors [1,10]. These fibroblast activation pathways have been identified as a common mechanism of fibrosis present across all tissues [1,3]. As anticipated, the anti-fibrotic efficacy of nintedanib was validated in various animal models of organ fibrosis, extending beyond its approved therapeutic indications [11,12,13,14]. As part of our multi-arm drug study on PTJC, we recently published data on the efficacy of nintedanib monotherapy, thereby closing the therapeutic gap between new antifibrotic drugs and conventional PTJC therapy [15]. A synergistic combination therapy targeting both the pro-inflammatory and pro-fibrotic response proved to be even more effective than an antifibrotic monotherapy in models of pulmonary fibrosis [16,17]. Hence, a pharmacological multi-level approach may also be more efficacious in suppressing the development of PTJC. Endothelin-1 (ET-1) has been implicated in the pathogenesis of inflammatory and fibrotic joint conditions, with elevated tissue levels of ET-1 associated with a greater fibrotic response [18,19,20,21,22]. The levels of this peptide hormone are regularly elevated in serum and synovial fluids in inflammatory joint diseases such as osteoarthritis or rheumatoid arthritis [21,23]. It plays a vital role in the acute inflammatory response, as it elicits the expression of pro-inflammatory interleukin-1 (IL-1), IL-6, and IL-8 along with tumor necrosis factor α (TNF- α) in monocytes, but also induces fibroblast activation and promotes the synthesis of extracellular matrix (ECM) [24,25]. Bosentan acts as a competitive antagonist that inhibits the binding of ET-1 on both ET_a_- and ET_b_ receptors [26,27].

To investigate the potential benefits of combination therapy with nintedanib and bosentan in post-traumatic joint contractures, we conducted a randomized, placebo-controlled study using a rat model following standardized knee trauma. The primary objective of this study was to ascertain whether the administration of nintedanib and bosentan was associated with improved knee joint mobility in our animal model of post-traumatic joint contracture. Secondly, we investigated the effect of combination therapy on histopathological changes in the posterior joint capsule and on pro-inflammatory and pro-fibrotic gene expression. The multi-arm study design is intended to further compare the efficacy of COMB therapy with that of nintedanib monotherapy. The data on nintedanib monotherapy had already been published previously [15].

Our findings will contribute to the development of preventive drug treatments for post-traumatic joint contracture and improve understanding of its underlying mechanisms.

## 2. Materials and Methods

### 2.1. Study Design

This investigation utilized 39 male Sprague Dawley rats at 11 weeks of age (Janvier Labs, Saint-Berthevin Cedex, France), weighing 444 ± 18 g on average. Animals were maintained individually under standard laboratory conditions, with a 12 h light/dark cycle at ambient temperature, and received a standard rodent diet and water without restriction. Three experimental groups were formed. One group received a combination of nintedanib and bosentan (COMB) (*n* = 13), another received only nintedanib, and a third group received a placebo (*n* = 13). The group size was calculated using a power analysis based on our preliminary work, carried out in collaboration with the Institute for Medical Biometry, Epidemiology, and Informatics at the University of Mainz. The primary endpoint was defined as the change in joint mobility (°). We used a mean difference of 10° and an equal standard deviation of 20°, with a Cohen’s d of 0.5. α was set at 0.05 and the power at 80%.

Each rat was assigned a four-digit subject number by the project administration. The randomization of subjects into the COMB, nintedanib, or placebo group, as well as the subsequent division into the qPCR and histology groups, was conducted utilizing GraphPad’s “random number generator” (https://www.graphpad.com/quickcalcs/randmenu/, accessed on 22 January 2023). The assignment of the subject number was kept confidential until data analysis. The animal caretakers and operating surgeons were unaware of the treatment administered to the animals and were not informed whether a drug or a placebo was used. The investigators conducting the histological and molecular biological examinations were only aware of the subject numbers.

**Figure 1:**
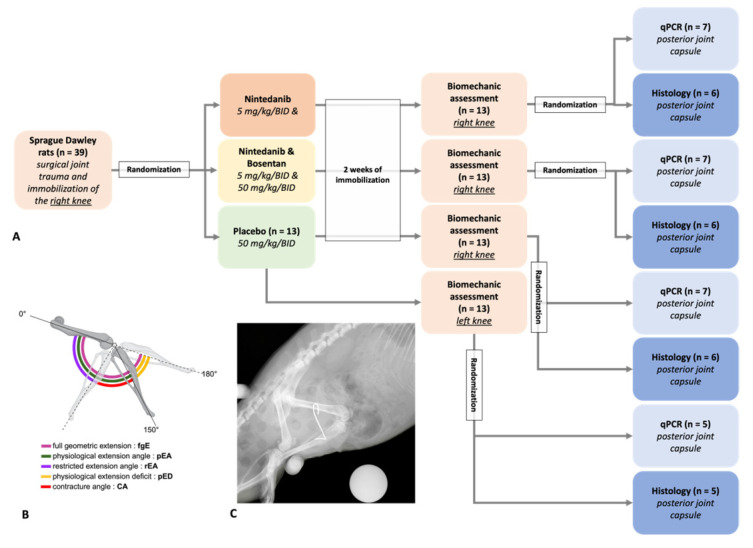
(**A**) Experimental design—animals randomized into three groups consisting of nintedanib and bosentan (COMB) (*n* = 13), nintedanib monotherapy (*n* = 13), or placebo (*n* = 13). All subjects (*n* = 39) received identical surgical trauma of the right knee immobilization. Following a 2-week immobilization period, biomechanical assessment was conducted, with posterior joint capsules randomly allocated for histological examination (*n* = 6) or gene expression analysis (*n* = 7). Uninjured left limbs from the placebo group (*n* = 13) provided reference values for the physiological knee (control). (**B**) Schematic illustration of the leg in a lateral view, showing different physiological and pathological joint angles. The extent of the contracture angle (CA) is used for comparison. CA was calculated using the following formula: CA = full geometric extension (fgE)—physiological extension deficit (pED)—restricted extension angle (rEA) (created in https://BioRender.com). (**C**) Lateral X-ray of the knee joint using K-wire arthrodesis at a 35° joint angle.

On experimental day 0, all subjects underwent a standardized protocol involving posterior knee capsule injury coupled with surgical joint immobilization via Kirschner wire (K-wire) placement in the right knee. Contralateral knees in the placebo cohort served as unmanipulated controls. From the first day of immobilization and continuing until sacrifice, animals received either nintedanib esylate (10 mg/kg daily divided into two 5 mg/kg oral doses, Boehringer Ingelheim International GmbH, Ingelheim am Rhein, Germany) and bosentan monohydrate (100 mg/kg daily divided into two 50 mg/kg oral doses; Janssen Pharmaceutica N.V., Beerse, Belgium), nintedanib esylate as a monotherapy (10 mg/kg daily divided into two 5 mg/kg oral doses) or a placebo (100 mg/kg daily divided into two 50 mg/kg oral doses; Winthrop Arzneimittel GmbH, Frankfurt am Main, Germany). The placebo contains lactose monohydrate, cellulose powder, magnesium stearate, and microcrystalline cellulose. All the drugs were ground into powder and blended with 0.25 g of white chocolate spread. This preparation was administered under supervision to ensure full ingestion. All drugs were administered at 12 h dosing intervals (Figure 1). The dosage of nintedanib was selected based on our previously published data on PTJC in a rat model and on the no-observed-adverse-effect level (NOAEL) identified from existing literature [15,28]. As there is currently no data available on the use of bosentan in the treatment of arthrofibrosis, the dosage is based on the antifibrotic effects observed in other rodent models of organ fibrosis [29,30].

Animals were excluded if they lost more than 20% body weight, showed abnormal behaviors like apathy or self-mutilation, had material failures (e.g., K-wire dislocation, material or bone fractures), or developed infections of the surgery site or wound healing disorders. Following two weeks of immobilization, all animals were sacrificed via carbon dioxide asphyxiation. The entire experimental protocol was conducted under double-blind conditions and received institutional ethics approval (ID 23 177-07/G 21-1-113).

### 2.2. Animal Model and Surgical Methodology

A joint injury was induced using our established protocol, as previously described in detail [15,31]. The experimental model incorporated posterior joint capsular disruption, hemarthrosis, intra-articular osseous damage, and temporary joint fixation. All surgical procedures were performed on the right hindlimb under sterile technique. Injury induction involved hyperextension of the right knee to 180° in anesthetized subjects, resulting in posterior joint capsule rupture. A standardized osseous defect (1.0 mm diameter × 3.0 mm depth) was created in the intercondylar region while preserving load-bearing cartilage surfaces and avoiding cruciate and collateral ligament damage. This intra-articular bone injury simulated an articular fracture and induced intra-articular hemorrhage. Subsequently, temporary fixation at a 35° joint angle was achieved using a bent K-wire spanning the femur and tibia for 14 days. The K-wire was secured through drill holes that traversed the diaphysis of both bones. Correct K-wire positioning, joint angle, and exclusion of inadvertent fractures were confirmed via lateral radiography (MX-20 Cabinet X-Ray System, Faxitron Bioptics, Tucson, AZ, USA). K-wire removal was performed at two weeks through the original surgical approach. Lateral radiography was repeated before removing the implant to rule out any fractures, material failure, or alterations in joint angle that may have occurred during the immobilization phase. General anesthesia was induced with 1% inhaled isoflurane, then maintained using subcutaneous administration of fentanyl (0.005 mg/kg), midazolam (4.0 mg/kg), and medetomidine (0.375 mg/kg). Anesthetic reversal was achieved with flumazenil (0.2 mg/kg) and atipamezole (1 mg/kg). Postoperative analgesia consisted of tramadol (1 mg/mL) added to drinking water, initiated three days preoperatively and continued for seven postoperative days. To minimize the effects of muscle tension on joint contracture development, periarticular myotomy was performed before all joint angle assessments, involving a skin incision and division of surrounding soft tissues 10 mm proximal and distal to the joint line.

### 2.3. Joint Angle Assessment

Joint angulation represents the femur-tibia angle measurement. “Full geometric extension” (fgE) occurs at 180° when femoral and tibial axes align. Since rats cannot achieve 180° extension, the difference between maximum extension at 35 Nmm torque in healthy rat knees and full geometric extension (180°) was designated as “physiological extension deficit” (pED) (Figure 1B). Therefore, healthy rat knees demonstrate no contracture but a physiological movement restriction by definition. Studies have shown that applying a torque of 35 Nmm enables full physiological extension in healthy knee joints while avoiding structural damage to the joint’s internal components [32,33]. Joint angle measurements were obtained from both intervention and placebo groups. Given that surgical procedures and immobilization affected right knees exclusively, left knees from the placebo cohort served as physiological references. The severity of knee joint contracture was quantified by calculating the contracture angle (CA) by determining the restricted extension angle (rEA) at 35 Nmm torque. The CA can therefore be calculated using the following formula:

CA = fgE − pED − rEA

All measurements were performed immediately post-sacrifice, two weeks following initial surgery. To evaluate the arthrogenic contracture component, complete skin and periarticular muscle resection was performed 1 cm from the femoral and tibial joint margins before measurement.

A custom automated mechanical testing apparatus was developed to evaluate rat knee contracture, as previously published. The arthrometer is based on previously described systems for testing the rabbit knee and rat elbow [34,35,36]. The system incorporated a linear motor slide (Elax Ex 50F20, Jenny Science AG, Rain, Switzerland) coupled with an actuator providing linear displacement and force measurement via Forceteq force-displacement technology (Jenny Science AG, Rain, Switzerland), controlled through a servo controller (XENAX Xvi Servo Controller with SMU Safety Motion Unit SS2, Jenny Science AG, Rain, Switzerland). Linear displacement was converted to rotational motion through rack and pinion gearing, enabling load-controlled rat knee flexion-extension testing. Following limb clamp securement, a single load cycle was performed on each joint, as multiple measurements could influence the PTJC. The cycle involved joint extension from the initial position to 180° extension. Force and torque were continuously monitored throughout the cycle. Force-displacement data were captured using Forceteq technology and converted to torque and angular position values. Knee angular position was calculated from the linear motor stroke position and transmitted to the control program. Maximum extension measurements were employed for contracture assessment. Both static and dynamic evaluations of the contracture were derived from the same load cycle. For the static evaluation, the joint angle corresponding to a torque of 35 Nmm during the load cycle was recorded.

### 2.4. Histological Tissue Processing

For histological evaluation, knee joints were carefully harvested immediately following sacrifice (*n* = 6 COMB group, *n* = nintedanib group, *n* = 6 placebo group, *n* = 5 unoperated control/left knee). Specimens were preserved in 4.5% neutral buffered formalin (Carl-Roth, Karlsruhe, Germany) for 48 h. Decalcification was subsequently performed using a tris(hydroxymethyl)aminomethane (TRIS)-buffered 17.7% ethylenediaminetetraacetic acid (EDTA) solution (Applichem, Darmstadt, Germany) for six weeks at 20 °C with continuous agitation using a roller mixer. Following paraffin embedding, five μm sagittal sections were obtained from central knee joint regions. Sections exhibiting tissue artifacts or structural damage to the meniscus, joint capsule, or synovial folds were excluded from analysis. A morphometric evaluation of quality was performed using hematoxylin and eosin (H&E) staining, in accordance with standard protocols [37]. Tissue section imaging and analysis were performed using ImageJ software (v. 1.54m). Two independent and blinded investigators performed histological evaluation. Cell quantification employed a standardized 62,500 μm^2^ area (250 μm × 250 μm), designated as the staining area or high-power field (HPF), adjacent to the posterior meniscal margin. Myofibroblast quantification utilized immunohistochemical α-smooth muscle actin (α-SMA) staining as described in our previous work [37]. α-SMA-positive (+) cells were differentiated based on vascular lumen proximity. α-SMA+ cells near vascular structures were classified as pericytes or vascular smooth muscle cells and excluded [38,39]. Remaining α-SMA+ positive cells were categorized as myofibroblasts. α-SMA-negative (−) cells within the extracellular matrix were classified as fibroblasts.

### 2.5. Quantitative PCR Tissue Processing

Posterior knee joint capsule segments (*n* = 7 COMB, *n* = nintedanib, *n* = 7 placebo group, *n* = 5 unoperated control/left knee) were immediately harvested post-sacrifice and immersed in RNAlater solution (Thermo Fisher Scientific, Waltham, MA, USA). Specimens were stored at −20 °C pending quantitative polymerase chain reaction (qPCR) processing. Ribonucleic acid (RNA) isolation involved manual sample grinding in liquid nitrogen, followed by additional homogenization using a Precellys homogenizer (Bertin Technologies, Montigny-le-Bretonneux, France) in TRIzol suspension (Thermo Fisher Scientific) according to the manufacturer’s specifications. Supernatants from centrifuged homogenates were subjected to a standard phenol-chloroform RNA extraction (Sigma-Aldrich, St. Louis, MO, USA). RNA samples were resuspended in nuclease-free water (Sigma-Aldrich) and quantified photometrically at 260 nm using a NanoDrop spectrophotometer (Thermo Fisher Scientific). Subsequently, 0.8 μg RNA per sample underwent reverse transcription to complementary DNA (cDNA) using M-MuLV reverse transcriptase, Random Primer Mix (New England Biolabs, Ipswich, MA, USA), and nucleotides (Bioron GmbH, Ludwigshafen, Germany). qPCR primers (Table 1) were designed from National Center for Biotechnology Information nucleotide sequences (https://www.ncbi.nlm.nih.gov/nucleotide/, accessed on 20 June 2023) using web-based primer design software (Eurofins Scientific, Luxembourg City, Luxembourg). qPCR reactions were performed on the qTOWER3 system (Jena Analytik, Jena, Germany) using Blue S’Green qPCR Master Mix (Biozyme Scientific GmbH, Hessisch Oldendorf, Germany) according to the manufacturer’s instructions. ΔCt (delta-cycle threshold) values were used for analysis, comparing *α-Sma*, *Il-6*, *Tgf-β*, *Nf-κB*, and *Ctgf* gene expression levels in posterior joint capsule tissues.

### 2.6. Statistical Analysis

Statistical analysis was performed using GraphPad Prism 10.3.1 (GraphPad Software, San Diego, CA, USA). Quantitative data presentation included bar charts, box plots displaying medians and quartiles, mean values ± standard deviation, and area under the curve (AUC) diagrams. Joint contracture angle analysis and expression analysis employed the Kruskal–Wallis test, while cell and vessel counts utilized one-way analysis of variance (ANOVA). The corresponding post hoc tests are noted in Section 3. A two-way ANOVA was used to analyze weight development. All gene expression measurements were conducted in triplicate, with statistical significance defined as *p* < 0.05. Box plot whiskers extended to the minimum and maximum values, lines indicated median values through boxes, and individual data points were represented by circles.

### 2.7. Graphic Design

The schematic illustrations were created using the BioRender platform (https://BioRender.com) or Microsoft PowerPoint V16.97.2 (Redmond, WA, USA).

## 3. Results

### 3.1. Weight Development and Study Attrition

**Figure 2:**
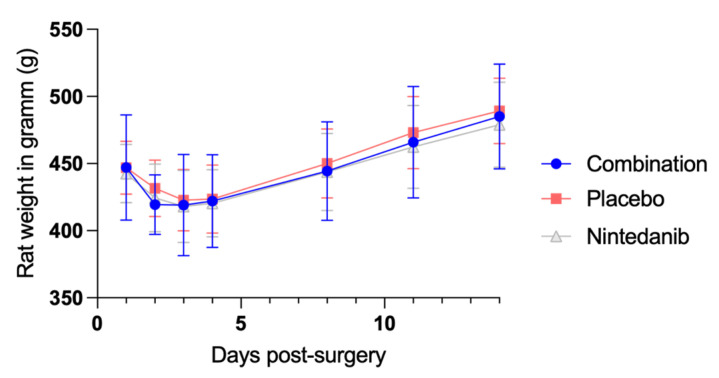
Weight development—no differences were observed between the COMB (*n* = 13), nintedanib (*n* = 13), and placebo (*n* = 13) groups over a two-week period (two-way ANOVA). The lowest weight was found across all three groups on the third postoperative day. Error bars indicate standard deviation.

The mean body weight of Sprague Dawley rats was 447 ± 39 g in the COMB group, 443 ± 22 g in the nintedanib group, and 446 ± 19 g in the placebo group at the inception of the experimental setup. On the third postoperative day, the lowest body weight was reached in both experimental groups. It differed significantly from the starting weight of the respective group but did not differ between the groups (COMB 419 ± 38 g, *p* ≤ 0.01; nintedanib 418 ± 27 g, *p* ≤ 0.01; placebo 422 ± 23 g, *p* ≤ 0.001; two-way ANOVA, F_time_ (2.959, 109) = 402.6, *p* < 0.0001, F_drug_ (2, 37) = 0.1757, *p* = ns, F_time(x)drug_ (5.971, 109) = 0.7333, *p* = ns). Subsequently, and throughout the experimental setup, there was a steady weight gain, culminating at 485 ± 39 g for the COMB group, 479 ± 32 g for the nintedanib group, and 489 ± 24 g for the placebo group, with no significant differences in weight gain between the groups. Postoperative weight loss did not result in the premature euthanasia of any rats (Figure 2).

One rat from the placebo group had to be removed from the arthrometric measurement, as X-ray examination before implant removal revealed a fracture proximal to the femoral insertion site. However, the posterior joint capsule of the specimen was subjected to histological and gene expression analysis due to the persistence of the surgically adjusted contracture angle. Similarly, another animal from the COMB group could not be used for arthrometry due to an incorrect preparation. Incomplete decalcification resulted in the exclusion of one sample from the COMB and placebo groups intended for histopathological examination. Two specimens from the control group designated for qPCR evaluation were ruled out because they were not stored in accordance with the protocol. No wound-healing disorders were observed.

### 3.2. Biomechanical Evaluation of the Joint Contracture

After the two-week immobilization period, the K-wire arthrodesis was surgically removed, and the extent of joint contracture was compared between the COMB and placebo groups. The calculated contracture angle (CA) was used for comparison. To this end, the restricted extension angle (rEA) was determined at 35 Nmm. The total contracture is calculated by subtracting the rEA from the full geometric extension (fgE = 180°). The full contracture consists of the CA and the physiological extension deficit (pED). The pED was evaluated on the physiological non-operated leg of the placebo group (control, *n* = 13) at a torque of 35 Nmm. On average, there was a pED of 30°. The CA is thus calculated as follows: CA = fgE (180°) − pED (30°) − rEA (Figure 1B). The immobilized knee joints of the placebo groups showed a significantly lower extension angle at 35 Nmm of torque compared to the non-immobilized knee joints (placebo 63.85° ± 11.1° vs. control 150.2° ± 20.5°; *p* < 0.0001, Welch’s test).

**Figure 3:**
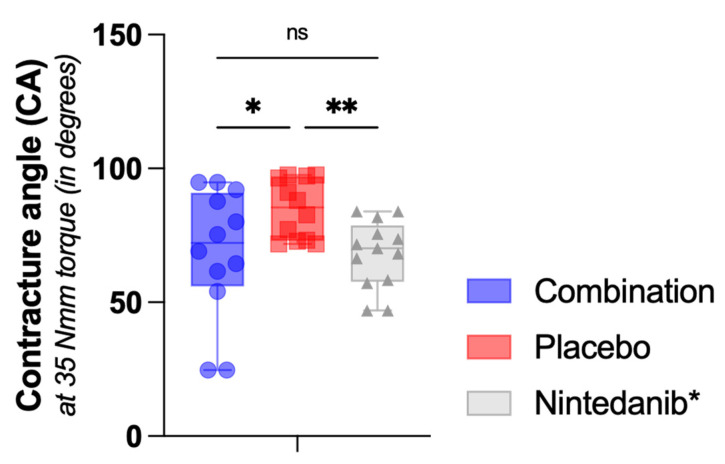
Biomechanic assessment at 35 Nmm torque—the combination group (*n* = 12) had a contraction angle 16.2° lower than the placebo group (*n* = 12) (* *p* ≤ 0.05). The contraction angle in the nintedanib group (*n* = 13) was 16.7° lower (** *p* ≤ 0.01) (* data from the nintedanib group has been published previously [15]). There was no statistically significant difference between the combination and nintedanib groups (ns; Kruskal–Wallis test). Error bars indicate minimum and maximum values.

A comparison of the CA of the COMB, nintedanib and placebo groups reveals a significant decrease in CA of 16.2° in the COMB group and 16.7° in the nintedanib* group with no significant difference between COMB and nintedanib groups two weeks after initial trauma (CA_comb_ = 68.6° ± 24.5 vs. CA_placebo_ = 84.8° ± 11.1°, *p* ≤ 0.05; CA_Nintedanib_ = 68.1 ± 12.7 vs. CA_placebo_ = 84.8° ± 11.1°, *p* ≤ 0.01; CA_comb_ vs. CA_Nintedanib_, ns.; Kruskal–Wallis test) (Figure 3) (* data from the nintedanib group has been published previously [15]).

The force required to move the joint from 60° to 120° was measured using dynamic arthrometry in the same measuring cycle. The force was quantified using the area under the curve. Though the total force required for extension was the lowest in the nintedanib group no statistical difference was achieved between groups (F_COMB_ = 3.8 ± 1.0 N vs. F_placebo_ = 3.8 ± 0.86 N, ns.; F_Nintedanib_ = 2.8 ± 0.6 N vs. F_placebo_ = 3.8 ± 0.86, ns.; F_COMB_ = 3.8 ± 1.0 N vs. F_Nintedanib_ = 2.8 ± 0.6 N, ns., Kruskal–Wallis test, post hoc two-stage step-up method of Benjamini, Krieger, and Yekutieli). For lower joint angles up to 90°, there was a tendency toward an advantage for combination or nintedanib* therapy (*p* = 0.07).

**Figure 4:**
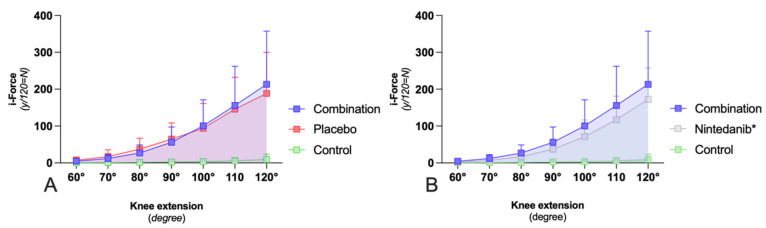
Dynamic biomechanic assessment. (**A**) Dynamic comparison of the contracture in the COMB (*n* = 12), placebo (*n* = 12), and control (*n* = 13) groups within the same measuring cycle. The whiskers indicate the standard deviation. COMB showed similar force values to those of the placebo group (Kruskal–Wallis test; post hoc two-stage step-up method of Benjamini, Krieger, and Yekutieli). In addition, the force required for non-operated and non-immobilized knee joints (control, *n* = 13) is shown. (**B**) Compares COMB (*n* = 12) with nintedanib (*n* = 13). No statistically significant difference was found between groups. To provide a clearer overview, each panel displayed three groups. A direct comparison between the nintedanib and placebo groups can be found in our previous publication [15].

Compared to healthy left knees in the control group, 24 times more force was required in the COMB and placebo group (p_COMB_ ≤ 0.01, p_placebo_ ≤ 0.01). In the nintedanib* group, the force required was 17 times higher (p_nintedanib_ ≤ 0.05). This confirms the presence of contracture in all three intervention groups (* data from the nintedanib group has been published previously [15]) (Figure 4).

### 3.3. Tissue Analysis of the Posterior Joint Capsule

#### 3.3.1. Alteration in Gene Expression in the Posterior Joint Capsule

**Figure 5:**
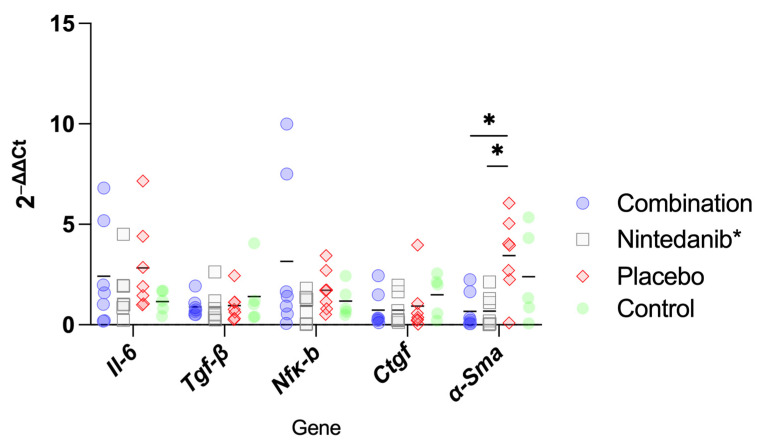
Profibrotic gene expression. The scatter plot compares the fold expression of *Il-6*, *Tgf-β*, *Nf-κb*, *Ctgf*, and α-Sma in the combination (*n* = 7), placebo (*n* = 7), and control (*n* = 5) groups. *Gapdh* was the reference gene. Statistical significance was marked with an asterisk (Kruskal–Wallis test, post hoc two-stage step-up method of Benjamini, Krieger, and Yekutieli). The whiskers indicate the minimum and maximum. (* Data from the nintedanib group has been published previously [15]).

Two weeks following surgical joint trauma, the expression levels of the upstream mediators *Il-6*, *Tgf-β*, *Nf-κb*, and *Ctgf*, as well as their downstream effector gene *α-Sma*, were analyzed in the posterior joint capsule. The expression levels of these mediators were normalized to the expression level of the housekeeping gene *Gapdh*. α-*Sma* expression levels were eight times lower in the COMB group and eleven times lower in the nintedanib* group than in the placebo group (Table 2, Kruskal–Wallis test, post hoc two-stage step-up method of Benjamini, Krieger, and Yekutieli). There is no significant difference between the COMB and nintedanib groups. There is also no statistical difference compared to the control group (ΔCt 12.92 ± 2.53; ns.). The upstream mediators did not show any measurable differences between the groups (Figure 4, Table 2) (* data from the nintedanib group has been published previously [15]) (Figure 5).

#### 3.3.2. Pathohistological Changes to the Posterior Joint Capsule

Compared to the placebo, the COMB group shows a significant reduction in absolute myofibroblast numbers in the localized sampling from the posterior joint capsule (*p* < 0.05, one-way ANOVA and post hoc Tukey test). The difference from the control group is even more pronounced (*p* < 0.001, one-way ANOVA and post hoc Tukey test). There is no statistically significant difference between the COMB, nintedanib, and control group (Table 3).

**Figure 6:**
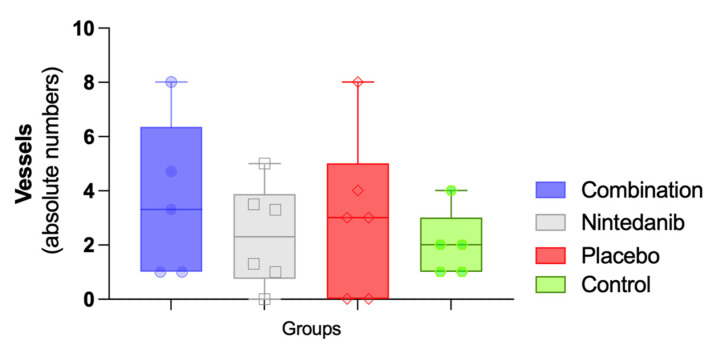
There was no statistically significant difference among the COMB (*n* = 5), nintedanib (*n* = 6), placebo (*n* = 6), or control (*n* = 5) groups (ns., one-way ANOVA with post hoc Tukey test).

The number of vessels was determined within the same high-power fields (HPFs). There was no significant difference between the groups (ns., one-way ANOVA with post hoc Tukey test) (Figure 6).

## 4. Discussion

The primary finding of this study is that combination therapy with bosentan and nintedanib did not yield statistically significant greater benefits than nintedanib alone in reducing early post-traumatic joint contracture (PTJC) in our rat model. Results demonstrated comparable, significant improvements in contracture angle, reductions in myofibroblast numbers, and decreased *α-Sma* expression relative to placebo. Our data indicate that targeting fibroblast activation with nintedanib is effective in mitigating PTJC development but that simultaneous endothelin receptor antagonism with bosentan does not enhance this effect under the tested conditions.

We have previously published data from this multi-arm pharmacological study on the measurable antifibrotic effect of nintedanib monotherapy, a multi-targeted tyrosine kinase inhibitor, in PTJC. Our results support the simultaneous inhibition of multiple receptors as an effective strategy for preventing the development of PTJC [15]. In contrast, inhibiting individual-specific cascades in PTJC models appears to have had little success. The most plausible explanation for the observed lack of effect appeared to be compensatory upregulation of pro-fibrotic mediators via bypass cascades. Similarly, selective inhibition of substance P, a neuropeptide found in elevated concentrations in arthrofibrotic joints, led to compensatory upregulation of IL-6 and IL-8, among other pro-fibrotic mediators, in Morrey’s contracture model [40]. Similarly, losartan’s and atorvastatin’s limited impact on the complex interplay of pro-fibrotic signaling cascades did not translate into a biomechanically measurable improvement in joint contracture. However, it did lead to a reduction in myofibroblasts in our preclinical PTJC setup [31,39,41]. However, nintedanib also reached its limits in treating PTJC. Even at a dose of 10 mg/kg/day (NOAEL) administered over a 14-day period, complete resolution of the joint contracture could not be achieved [15,28].

Although nintedanib emerges as a promising therapeutic agent for PTJC, the drug may, in theory, impair postoperative wound healing and increase the risk of intraoperative bleeding at higher doses [42]. It should be noted that no complications related to wound healing occurred during treatment with nintedanib.

Using an anti-inflammatory therapeutic agent in conjunction with nintedanib has the potential to enhance therapeutic efficacy and mitigate deleterious side effects associated with increased dosage. A potential therapeutic agent for use in combination with nintedanib could be bosentan, an endothelin-1 receptor inhibitor (ET-1). Preclinical and clinical findings suggest a critical role for endothelin-1 (ET-1) in the development of various types of fibrosis [21,23,26,43,44].

This part of the multi-arm study investigates the efficacy of concurrent administration of bosentan and nintedanib in preventing early post-traumatic joint contracture (PTJC) formation in surgically traumatized knee joints of 39 male Sprague Dawley rats over a two-week period. The primary outcome was an improved contracture angle as measured by arthrometry.

Using our PTJC model, we previously demonstrated that the majority of joint contracture already develops at the biomechanical level after a two-week immobilization phase [39,41]. Accordingly, all intervention groups (COMB, nintedanib, and placebo) showed significantly greater contracture of the operated and immobilized leg than the non-immobilized leg in the placebo group (control). This early development of joint contracture in PTJC is consistent with the findings of Kaneguchi et al., who observed that the majority of arthrogenic contracture also developed after a comparable period of immobilization following anterior cruciate ligament reconstruction in their rat model [45]. This coincides with the time when myofibroblast differentiation is most pronounced, thus allowing for the most reliable determination of the influence of drug intervention on myofibroblasts, the primary effector cell of PTJC [41,46,47].

Direct comparison of the intervention groups in this study suggests that the COMB partially mitigates PTJC formation compared with placebo. However, no statistically significant superiority over nintedanib monotherapy was observed (Figure 3).

Compared to the placebo group, it led to a significant decrease in the absolute and relative number of myofibroblasts and a reduction in α-SMA expression levels, though the results did not differ substantially from those of the nintedanib group.

The lack of improved therapeutic efficacy could be attributed to the inadequate bioavailability of the two drugs. The absorption and bioavailability of nintedanib are reduced due to the effect of the P-glycoprotein transporter. Bosentan is associated with the induction of this enzyme and could therefore reduce the plasma concentration of nintedanib [48,49]. A pharmacokinetic study examining the simultaneous administration of the two drugs over seven days in 13 healthy male subjects showed no significant effect on the plasma concentration of nintedanib. However, potential drug–drug interactions cannot be ruled out over a more extended intervention period [49].

A synergistic effect on *Ctfg*, a profibrotic mediator, resulting from direct inhibition of its receptor by nintedanib and, indirectly, from reduced *Ctgf* expression by bosentan, could also have been converted into a redundant mechanism of action [10,50]. However, two weeks after the trauma, *Ctgf* levels in both intervention groups were already comparable to those in the control group, which could indicate that *Ctgf* expression may be elevated at an earlier stage of PTJC.

Furthermore, gene expression analysis demonstrated that other key inflammatory mediators, such as *Il-6* and *Nf-κb*, exhibited comparable levels across all groups following the two-week period. This finding may be indicative of a subsided acute inflammatory response. Morrey et al. also arrived at the same conclusion in their work on the molecular landscape of post-traumatic arthrofibrosis in a rabbit model. Microarray analysis of 380 genes confirmed that the majority of proinflammatory genes had normalized after two weeks [18]. Earlier sampling of *Ctgf*, *Il-6*, and *Nf-κB* in PTJC could serve to confirm an earlier peak in these mediators.

Some limitations of our study should be noted. No data on the use of bosentan in PTJC treatment is currently available. The dosage is based on its antifibrotic effects in other rodent models of organ fibrosis [29,30]. No values are available for the maximum plasma concentration (C_(max)) or the time to steady state for the selected dosage. Serum and synovial fluid concentrations of the drugs were not determined. Furthermore, ET-1 activity was not directly assessed. The small group size in the histopathological parameter and gene expression analyses could have contributed to the variability observed in the data, potentially masking significant differences. Assessing proinflammatory gene expression at a specific point in time does not provide a complete picture of the importance of selected mediators on PTJC or the influence of drug therapy. The inclusion of earlier and later time points in a future study design will allow for a more accurate reflection of the temporal relationship between drug exposure, the pro-inflammatory phase, fibrotic remodeling processes, and biomechanics. Forced extension of the fibrotic knee joints resulted in tearing of the capsule, making morphometric evaluation of the capsular length and diameter unfeasible. For this reason, histological analysis was only performed locally in the area of the joint capsule adjacent to the meniscus base. The alterations measured can thus only be applied to the entire capsule to a limited extent. Lastly, immunohistochemistry was only performed on myofibroblasts.

In terms of practical application, the results highlight the potential of the antifibrotic agent nintedanib as a promising option for the pharmacological treatment of early PTJC. A detailed evaluation of the temporal dynamics of the various mediators involved in the PTJC could help in developing an optimal dosing profile for the active ingredients. At the same time, other routes of administration, such as intra-articular injection in animal models, should also be investigated in order to minimize systemic side effects and maximize local drug concentrations without potentially impairing wound healing. This could eventually lead to the development of prophylactic therapy for PTJC in cases of prolonged joint immobilization and in patients at risk.

## 5. Conclusions

Combination therapy with bosentan and nintedanib (COMB) and nintedanib monotherapy resulted in a significant reduction in post-traumatic joint contracture (PTJC) compared with placebo. Each treatment group showed a significant reduction in contracture angles, with no significant differences between groups. Histological analysis showed a significant decrease in myofibroblast numbers and α-SMA expression in both the COMB and nintedanib groups compared with placebo, though, as before, there were no differences between the two interventions. In both groups, there were no significant changes in the gene expression of upstream profibrotic markers after two weeks.

Overall, these results suggest that combining nintedanib with bosentan offers no additional benefit in preventing early PTJC under the tested conditions. Therefore, nintedanib monotherapy remains a potentially effective treatment option. The optimal application form, duration, and dosage must be investigated in future studies.

## Figures and Tables

**Table 1:** Gene targets and primer sequences utilized for quantitative PCR analysis.

Gene	Primer	Sequence
*Gapdh*	Forward	AACGACCCCTTCATTGACCT
	Reverse	CCCCATTTGATGTTAGCGGG
*α-Sma*	Forward	CATCATGCGTCTGGACTTGG
	Reverse	CCAGGGAAGAAGAGGAAGCA
*Il-6*	Forward	CCACCCACAACAGACCAGTA
	Reverse	ACTCCAGAAGACCAGAGCAG
*Tgf-β*	Forward	CCCTACATTTGGAGCCTGGA
	Reverse	CGCACGATCATGTTGGACAA
*Nf-κb*	Forward	AGAGGATGTGGGGTTTCAGG
	Reverse	GCTGAGCATGAAGGTGGATG
*Ctgf*	Forward	TCCCAAAATCTCCAAGCCTA
	Reverse	GTAATGGCAGGCACAGGTCT

**Table 2:** The ΔCt-values of *Il-6*, *Tgf-β*, *Nf-κb*, *Ctgf*, and *α-Sma* in the COMB (*n* = 7), nintedanib* (*n* = 7), placebo (*n* = 7), and control (*n* = 5) groups are shown (means and standard deviation). *Gapdh* was used as the reference gene (* data from the nintedanib group has been published previously [15]).

Gene	Combination	Nintedanib*	Placebo	Control
	Mean ± SD	Mean ± SD	Mean ± SD	Mean ± SD
*Il-6*	9.33 ± 2.07	9.37 ± 1.35	9.57 ± 0.83	9.57 ± 0.83
*Tgf-β*	8.17 ± 0.67	8.46 ± 1.16	7.95 ± 1.40	7.95 ± 1.40
*Nf-κb*	7.11 ± 2.38	8.63 ± 2.5	6.96 ± 0.96	7.51 ± 0.94
*Ctgf*	6.46 ± 1.62	6.34 ± 2.51	6.50 ± 2.13	5.12 ± 1.59
*α-Sma*	15.04 ± 2.18	15.42 ± 3.14	11.95 ± 2.13	12.92 ± 2.53

**Table 3:** Total amount and ratio of myofibroblasts. Myofibroblasts are significantly reduced in the COMB (*n* = 5; *, *p* < 0.05), nintedanib* (*n* = 6; ns.) control group (*n* = 5; **, *p* < 0.01) compared to the placebo group (*n* = 6; one-way ANOVA with post hoc Tukey test) (* data from the nintedanib group has been published previously [15]).

Cells/ Groups	Myofibroblasts No. Cells/HPF ± SD	Total Cells No. Cells/HPF ± SD	Ratio Myofibroblasts/ Total Cells ± SD
COMB	9 ± 6 *	70 ± 29	12.9 ± 10.1%
Nintedanib*	12 ± 9	118 ± 34	10.3 ± 8.2%
Placebo	20 ± 12	83 ± 47	24.1 ± 21.0%
Control	1 ± 1 **	78 ± 38	1.2 ± 1.5%

## Data Availability

The datasets generated and analyzed during the current study are available from the corresponding author upon reasonable request.

## Abbreviations

The following abbreviations are used in this manuscript:

ANOVA	analysis of variance
AUC	area under the curve
CA	contracture angle
CI	confidence interval
COMB	combination therapy
CTGF	connective tissue growth factor
ECM	extracellular matrix
ET-1	endothelin-1
ETR	endothelin receptor
fgE	full geometric extension
FGF	fibroblast growth factor
GAPDH	glyceraldehyde-3-phosphate dehydrogenase
HPF	high-power field
IL	interleukin
JA	joint angle
K-wire	Kirschner wire
ns.	not significant
NF-κB	nuclear factor ‘kappa-light-chain-enhancer’ of activated B-cells
NOAEL	no-observed-adverse-effect level
PCR	polymerase chain reaction
PDGF	platelet-derived growth factor
pEA	physiological extension angle
pED	physiological extension deficit
PTJC	post-traumatic joint contracture
rEA	restricted extension angle
RNA	ribonucleic acid
TGF-β	transforming growth factor beta
TNF-α	tumor necrosis factor-α
VEGF	vascular endothelial growth factor
α-SMA	alpha-smooth muscle actin
